# Dissociating motor impairment from five-choice serial reaction time task performance in a mouse model of Angelman syndrome

**DOI:** 10.3389/fnbeh.2022.968159

**Published:** 2022-09-23

**Authors:** Paola N. Negrón-Moreno, David T. Diep, Caleigh D. Guoynes, Michael S. Sidorov

**Affiliations:** ^1^University of Puerto Rico-Cayey, Cayey, PR, United States; ^2^Department of Cell Biology and Physiology, Neuroscience Center, University of North Carolina at Chapel Hill, Chapel Hill, NC, United States; ^3^University of Maryland, College Park, College Park, MD, United States; ^4^Center for Neuroscience Research, Children’s National Medical Center, Washington, DC, United States; ^5^Departments of Pediatrics and Pharmacology and Physiology, The George Washington University School of Medicine and Health Sciences, Washington, DC, United States

**Keywords:** Angelman syndrome, behavior, attention, motor, five-choice serial reaction time task

## Abstract

Angelman syndrome (AS) is a single-gene neurodevelopmental disorder associated with cognitive and motor impairment, seizures, lack of speech, and disrupted sleep. AS is caused by loss-of-function mutations in the *UBE3A* gene, and approaches to reinstate functional *UBE3A* are currently in clinical trials in children. Behavioral testing in a mouse model of AS (*Ube3a*^*m–/p+*^) represents an important tool to assess the effectiveness of current and future treatments preclinically. Existing behavioral tests effectively model motor impairments, but not cognitive impairments, in *Ube3a*^*m–/p+*^ mice. Here we tested the hypothesis that the 5-choice serial reaction time task (5CSRTT) can be used to assess cognitive behaviors in *Ube3a*^*m–/p+*^ mice. *Ube3a*^*m–/p+*^ mice had more omissions during 5CSRTT training than wild-type littermate controls, but also showed impaired motor function including open field hypoactivity and delays in eating pellet rewards. Motor impairments thus presented an important confound for interpreting this group difference in omissions. We report that despite hypoactivity during habituation, *Ube3a*^*m–/p+*^ mice had normal response latencies to retrieve rewards during 5CSRTT training. We also accounted for delays in eating pellet rewards by assessing omissions solely on trials where eating delays would not impact results. Thus, the increase in omissions in *Ube3a*^*m–/p+*^ mice is likely not caused by concurrent motor impairments. This work underscores the importance of considering how known motor impairments in *Ube3a*^*m–/p+*^ mice may affect behavioral performance in other domains. Our results also provide guidance on how to design a 5CSRTT protocol that is best suited for future studies in *Ube3a* mutants.

## Introduction

Angelman syndrome (AS) is a rare neurodevelopmental disorder characterized by cognitive and motor impairment, lack of speech, seizures, abnormal EEG patterns, disrupted sleep, short attention span, and a signature behavioral profile that includes hypersociability (Angelman, 1965; Thibert et al., 2013; Bird, 2014; Buiting et al., 2016). Cognitive impairment and motor dysfunction are among the most common features of AS, both affecting nearly all individuals (Thibert et al., 2013). AS is caused by loss-of-function mutations in the maternally inherited *UBE3A* gene, which encodes UBE3A protein, an E3 ubiquitin ligase involved in regulating protein degradation (Kishino et al., 1997; Lee et al., 2014; Bonello et al., 2017). No effective treatment currently exists for AS, but approaches to unsilence the dormant paternal *UBE3A* allele have been successful in mouse models (Huang et al., 2011; Meng et al., 2015; Lee et al., 2018; Wolter et al., 2020; Elgersma and Sonzogni, 2021; Milazzo et al., 2021; Schmid et al., 2021) and are now in clinical trials in children (Copping et al., 2021).

The widely used *Ube3a*^*m–/p+*^ mouse model (Jiang et al., 1998) recapitulates some of the most common features of AS, including locomotor dysfunction, seizures, abnormal EEG, and sleep impairments (Jiang et al., 1998; Colas et al., 2005; Heck et al., 2008; Allensworth et al., 2011; Ehlen et al., 2015; Shi et al., 2015; Born et al., 2017; Sidorov et al., 2017; Sonzogni et al., 2018; Rotaru et al., 2020; Copping and Silverman, 2021). Mouse behavior has provided a valuable readout to demonstrate the preclinical effectiveness of paternal *Ube3a* unsilencing and other treatment strategies (van Woerden et al., 2007; Daily et al., 2011; Meng et al., 2015; Sonzogni et al., 2020; Wolter et al., 2020; Milazzo et al., 2021; Schmid et al., 2021). However, cognitive impairment has proven more difficult to model in *Ube3a*^*m–/p+*^ mice. Prefrontal cortex is critical for executive function and cognitive control in humans, and impaired prefrontal structure and function has been observed in individuals with autism and other neurodevelopmental and neuropsychiatric disorders (Miller and Cohen, 2001; O’Hearn et al., 2008; Solomon et al., 2014, 2016). Expanding the suite of *Ube3a*^*m–/p+*^ behavioral testing to include complex, prefrontally-encoded tasks will enable a wider assessment of the effectiveness of treatments. Recent evidence suggests that loss of *Ube3a* results in circuit-level impairments in mice in two prefrontal subregions: infralimbic cortex and anterior cingulate cortex (ACC) (Rotaru et al., 2018; Sidorov et al., 2018, 2020). We previously demonstrated that infralimbic circuit dysfunction in *Ube3a*^*m–/p+*^ mice can be assessed behaviorally using an operant extinction task (Sidorov et al., 2018). Here, we tested the hypothesis that attentional behavior, regulated in part by ACC circuits, would be impaired in *Ube3a*^*m–/p+*^ mice.

The five-choice serial reaction time task (5CSRTT) is a commonly used behavioral test for assessing attention and impulsivity in rodents (Robbins, 2002; Asinof and Paine, 2014; Higgins and Silenieks, 2017). Briefly, food restricted mice are trained to respond to a light cue with a nosepoke to receive a food reward. The light cue has a fixed short duration, and the number of trials omitted (“omissions”) provides a readout of attention, while the number of premature responses during an intertrial interval provides a readout of impulsivity. Lesion studies and chemogenetic manipulations have demonstrated that rodent ACC regulates attentional performance during the 5CSRTT (Chudasama et al., 2003; Koike et al., 2016; Norman et al., 2021). The 5CSRTT has been widely used in rodent models of a variety of neurodevelopmental and neuropsychiatric disorders, including autism, addiction, and attention deficit hyperactivity disorder (Kramvis et al., 2013; Lloyd et al., 2013; Dommett, 2014; Anshu et al., 2017; Caballero-Puntiverio et al., 2017; Justinussen et al., 2020).

Here we report that *Ube3a*^*m–/p+*^ mice have both increased omissions and motor impairments during the 5CSRTT. Gross motor hypoactivity in *Ube3a*^*m–/p+*^ mice does not drive the change in omissions. However, *Ube3a*^*m–/p+*^ mice take longer to eat pellet rewards, confounding interpretations of omissions on adjacent trials. By evaluating only non-adjacent trials, we are able to successfully disassociate omissions from potential motor confounds. This study demonstrates the need to carefully account for motor impairments in *Ube3a*^*m–/p+*^ mice when assessing complex behavior.

## Results

### *Ube3a*^*m–/p+*^ mice are hypoactive

**FIGURE 1:**
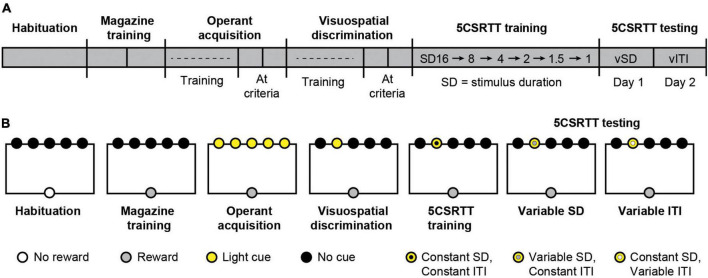
The five-choice serial reaction time task (5CSRTT) measures attention and impulsivity in mice. **(A)** Timeline of 5CSRTT. vSD, variable stimulus duration; vITI, variable intertrial interval. **(B)** Schematic of cues and rewards across all stages of 5CSRTT.

**FIGURE 2:**
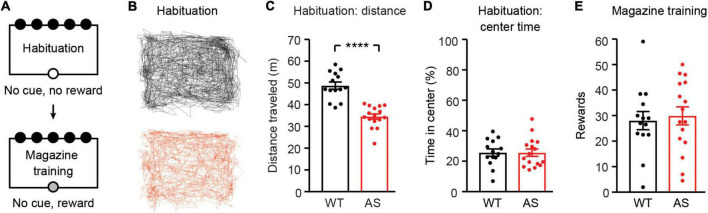
*Ube3a*^*m–/p+*^ mice are hypoactive during habituation and receive normal rewards during magazine training. WT: black, *n* = 14. *Ube3a*^*m–/p+*^ (AS): red, *n* = 16. **(A)** Schematic of habituation and magazine training phases. **(B)** Example paths of individual WT and *Ube3a*^*m–/p+*^ mice during habituation. **(C)** Distance traveled and **(D)** time spent in the center of the arena during habituation. **(E)** Average rewards received across 2 days of magazine training. ****p< 0.0001. Error bars indicate ± SEM.

We tested the performance of *Ube3a*^*m–/p+*^ mice and wild-type (WT) littermates on the 5CSRTT (Figure 1). Prior to training, food restricted mice were first habituated to the behavioral chamber for one session (Figure 2A). During habituation, *Ube3a*^*m–/p+*^ mice were less active than WT littermates [Figures 2B,C; *t*_(28)_ = 6.971, *p*< 0.0001] but spent similar amounts of time in the center of the chamber [Figure 2D; *t*_(28)_ = 0.01024, *p* = 0.9919]. Open field hypoactivity in *Ube3a*^*m–/p+*^ mice is expected and has been reported by many groups (Allensworth et al., 2011; Huang et al., 2013; Born et al., 2017; Sonzogni et al., 2018). Following habituation, mice underwent 2 days of magazine training, where pellet rewards were delivered upon every nosepoke into the illuminated food magazine. WT and *Ube3a*^*m–/p+*^ mice did not differ in the amount of rewards received during this unrestricted phase [Figure 2E; *t*_(28)_ = 0.3704, *p* = 0.7139], suggesting no gross difference in motivation between groups.

### *Ube3a*^*m–/p+*^ mice show expected impairments in operant acquisition and visuospatial discrimination

**FIGURE 3:**
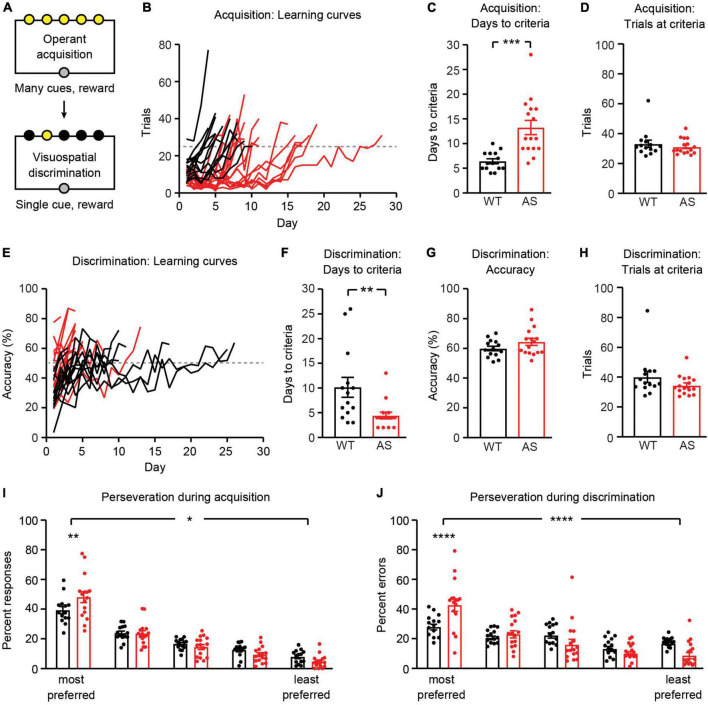
*Ube3a*^*m–/p+*^ mice have abnormal performance during operant acquisition and visuospatial discrimination. WT: black, *n* = 14. *Ube3a*^*m–/p+*^ (AS): red, *n* = 16. **(A)** Schematic of operant acquisition and visuospatial discrimination phases. **(B)** Learning curves during operant acquisition; each line represents one mouse and dotted line represents 25 trial per session threshold. **(C)** Days to reach operant acquisition criteria (>25 trials per session on two consecutive days). **(D)** Average trials per session on two finals days of operant acquisition, when mice have reached criteria. **(E)** Learning curves during visuospatial discrimination. Dotted line represents 50% accuracy threshold. **(F)** Days to reach visuospatial discrimination criteria (>25 trials per session and >50% accuracy on two consecutive days). **(G)** Average accuracy on two final days of visuospatial discrimination (at criteria). **(H)** Average trials per session on two final days of visuospatial discrimination (at criteria). **(I)** Perseveration during operant acquisition: nosepokes are rank-ordered by preference. **(J)** Perseveration during visuospatial discrimination. *p < 0.05, **p < 0.01, ***p< 0.001, ****p< 0.0001. Error bars indicate ± SEM.

Following magazine training, mice underwent operant acquisition and visuospatial discrimination training (Figure 3A). During operant acquisition, a nosepoke into any of five illuminated apertures resulted in reward. During visuospatial discrimination, a nosepoke into one illuminated aperture (that varied each trial) resulted in reward. Operant acquisition and visuospatial discrimination stages were considered complete when mice reached pre-determined performance criteria (see section “Materials and methods”). *Ube3a*^*m–/p+*^ mice took longer than WT littermates to complete operant acquisition training (Figures 3B,C; *t*_(28)_ = 4.251, *p* = 0.0002). There was no difference between groups in the amount of trials per session during the final 2 days of acquisition [Figure 3D; *t*_(28)_ = 0.7584, *p* = 0.4545]. *Ube3a*^*m–/p+*^ mice completed visuospatial discrimination training in fewer sessions than WT controls [Figures 3E,F; *t*_(28)_ = 2.840, *p* = 0.0083]. *Ube3a*^*m–/p+*^ mice completed visuospatial discrimination training faster than WT littermates because they had fewer incorrect responses and a similar amount of correct responses (Supplementary Figure 1), resulting in an increased accuracy (Figure 3E). However, their performance once they reached criteria was not statistically different: mice in both groups averaged ∼60–65% accuracy [criteria = 50%; Figure 3G; *t*_(28)_ = 1.555, *p* = 0.1312] and had a similar number of trials per session [Figure 3H; *t*_(28)_ = 1.451, *p* = 0.1580].

Operant acquisition and the transition from acquisition to discrimination provided an opportunity to assess perseveration in *Ube3a*^*m–/p+*^ mice. During operant acquisition, we measured the number of nosepokes into each of five illuminated apertures and rank-ordered the apertures from most preferred to least preferred (by number of nosepokes) for each mouse. *Ube3a*^*m–/p+*^ mice were more likely to nosepoke into preferred apertures [Figure 3I; genotype X aperture interaction: *F*_(4,__140)_ = 3.424, *p* = 0.0105]. In addition, in *Ube3a*^*m–/p+*^ mice, errors during visuospatial discrimination were more commonly made in apertures where animals demonstrated a preference during acquisition [Figure 3J; genotype X aperture interaction: *F*_(4,__140)_ = 7.143, *p* < 0.0001]. Together, these results suggest that *Ube3a*^*m–/p+*^ mice have increased perseveration and are consistent with our previous study of visuospatial discrimination in *Ube3a*^*m–/p+*^ mice (Sidorov et al., 2018).

### *Ube3a*^*m–/p+*^ mice have increased omissions during five-choice serial reaction time task training

**FIGURE 4:**
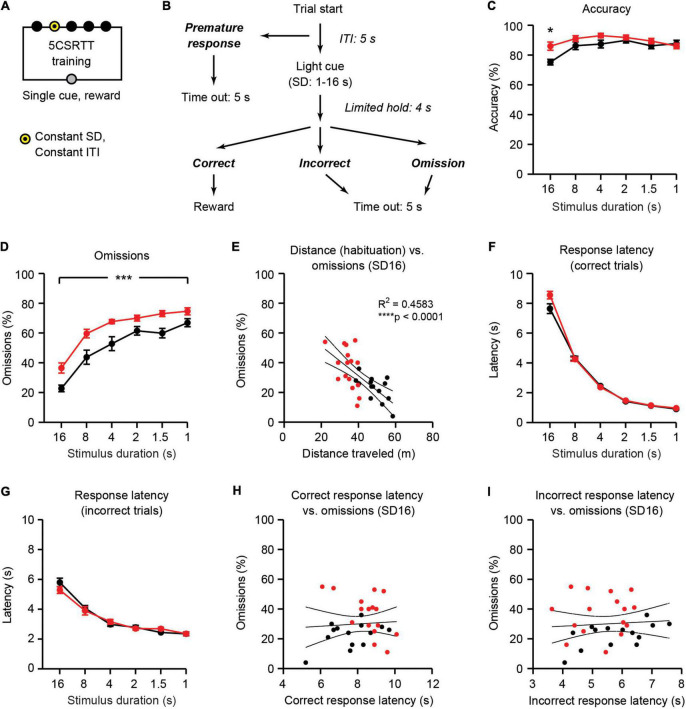
*Ube3a*^*m–/p+*^ mice have increased omissions and motor impairments during 5CSRTT training. WT: black, *n* = 14. *Ube3a*^*m–/p+*^ (AS): red, *n* = 16. **(A)** Schematic of 5CSRTT training. SD: stimulus duration, ITI: intertrial interval. **(B)** Trial structure for individual trials (adapted from Asinof and Paine, 2014). **(C)** Accuracy [correct/(correct + incorrect)] on the final session at each stimulus duration. **(D)** Omissions during 5CSRTT training are increased in *Ube3a*^*m–/p+*^ mice. **(E)** Distance traveled during habituation is negatively correlated with omissions. **(F)** Response latency on correct trials. **(G)** Response latency on incorrect trials. **(H)** Correct response latency and **(I)** incorrect response latency are not correlated with omissions. *p < 0.05, ***p< 0.001, ****p< 0.0001. Error bars indicate ± SEM.

5CSRTT training was similar to visuospatial discrimination in that only one target was illuminated per trial. However, during 5CSRTT training, the target was illuminated only for a fixed stimulus duration (Figure 4A). Thus, in addition to correct and incorrect responses, 5CSRTT trials could also result in two additional outcomes: omissions and premature responses (Figure 4B). Omissions were defined when mice did not respond during either the light cue or during a 4 s limited hold period immediately following light presentation. Premature responses were defined when mice responded during a 5 s intertrial interval prior to light cue. The stimulus duration was constant within each session, and it gradually decreased from 16 to 1 s across sessions, as mice reached pre-determined performance criteria (see section “Materials and methods”). Both *Ube3a*^*m–/p+*^ mice and WT mice performed the task with high accuracy (typically > 80%) across all stimulus durations tested (Figure 4C). Accuracy in *Ube3a*^*m–/p+*^ mice was statistically higher than WT littermates [main effect of genotype: *F*_(1,__28)_ = 5.077, *p* = 0.0323]. *Post-hoc* tests revealed that the small overall increase in accuracy in *Ube3a*^*m–/p+*^ mice was significant only at a stimulus duration of 16 s (*p* = 0.0196). The increase in accuracy in *Ube3a*^*m–/p+*^ mice at the beginning of 5CSRTT training is consistent with the trend toward increased accuracy seen at the end of visuospatial discrimination (Figure 3G) and is driven by *Ube3a*^*m–/p+*^ mice having fewer incorrect trials (Supplementary Figure 2).

As expected, omissions increased across 5CSRTT training as the stimulus duration decreased [Figure 4D; main effect of stimulus duration: *F*_(5,__140)_ = 105.1, *p* < 0.0001]. *Ube3a*^*m–/p+*^ mice had more omissions than WT littermates across the duration of 5CSRTT training [Figure 4D; main effect of genotype: *F*_(1,__28)_ = 14.55, *p* = 0.0007]. There was no interaction between genotype and stimulus duration [*F*_(5,__140)_ = 1.317, *p* = 0.2602]. A trend toward increased omissions was also observed in *Ube3a*^*m–/p+*^ mice during both 5CSRTT testing phases (Supplementary Figure 3). Impulsivity, defined as the number of premature responses during the intertrial interval, was normal in *Ube3a*^*m–/p+*^ mice during 5CSRTT testing (Supplementary Figure 4).

### Hypoactivity in *Ube3a*^*m–/p+*^ mice confounds measurement of omissions

Increased omissions in *Ube3a*^*m–/p+*^ mice during 5CSRTT training are difficult to interpret because of the potential confound of hypoactivity (Figure 2C). Indeed, distance traveled during habituation was negatively correlated with omissions during 5CSRTT training (Figure 4E; *R*^2^ = 0.4583, *p*< 0.0001). We reasoned that if hypoactivity is the underlying cause of increased omissions in *Ube3a*^*m–/p+*^ mice, this hypoactivity would be observed both during habituation and during motivated 5CSRTT training sessions. Therefore, we assessed the response latency during correct and incorrect trials during 5CSRTT training. Response latency was defined as the time from light cue to either correct response in the cued aperture or incorrect response in a dark aperture. Response latency on both correct trials and incorrect trials was not different between WT and *Ube3a*^*m–/p+*^ mice [Figures 4F,G; main effect of genotype: *F*_(1,__28)_ = 3.378, *p* = 0.0767 for correct, *F*_(1,__28)_ = 0.2395, *p* = 0.6284 for incorrect]. In addition, neither correct nor incorrect response latency was correlated with omissions within sessions (Figures 4H,I; *R*^2^ = 0.004682, *p* = 0.7194 for correct, *R*^2^ = 0.006026, *p* = 0.6835 for incorrect). Together, these results suggest that it is unlikely that gross motor impairments are the acute cause of increased omissions: at each stimulus duration tested, *Ube3a*^*m–/p+*^ mice are able to reach the illuminated aperture in the same amount of time as WT littermates.

### Increased omissions in *Ube3a*^*m–/p+*^ mice are driven partially, but not fully, by delays in eating rewards from previous trials

Hypoactivity does not seem to be the acute cause of increased omissions in *Ube3a*^*m–/p+*^ mice (Figures 4F–I). However, the strong negative correlation between hypoactivity during habituation and omissions during 5CSRTT training (Figure 4E) motivated us to consider other potential confounds in *Ube3a*^*m–/p+*^ mice. When observing video recordings of sessions, we noticed that during some omissions, mice were not attending to the stimulus because they had not yet finished eating the reward pellet from the previous trial. We reasoned that if *Ube3a*^*m–/p+*^ mice have delays in eating rewards, then this might account for some or all of the increase in omissions observed. Therefore, we asked two related questions: (a) are omissions more common following correct trials, and (b) do *Ube3a*^*m–/p+*^ mice take longer to eat pellet rewards?

**FIGURE 5:**
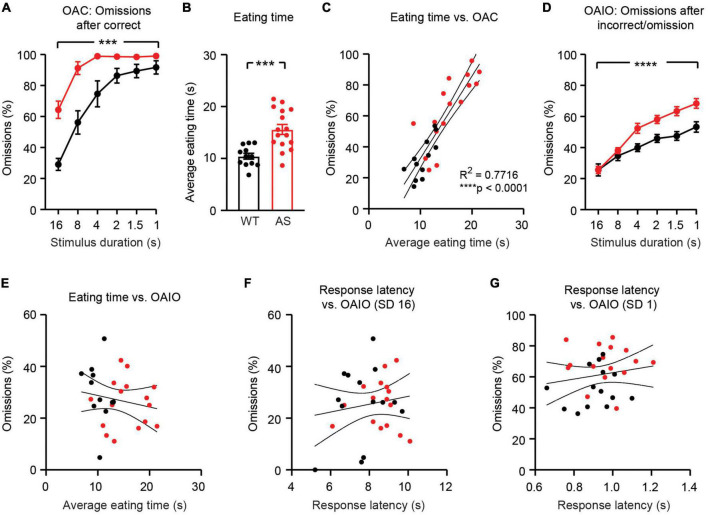
Increased omissions persist in *Ube3a*^*m–/p+*^ mice after controlling for motor confounds. WT: black, *n* = 14 (except *n* = 12 in **(B)**; two videos were corrupted and not analyzed). *Ube3a*^*m–/p+*^ (AS): red, *n* = 16. **(A)** Omissions after correct responses (OAC) are greater in *Ube3a*^*m–/p+*^ mice. **(B)** Eating time is greater in *Ube3a*^*m–/p+*^ mice. **(C)** Eating time is tightly correlated with OAC. **(D)** Omissions after non-correct trials (OAIO) are greater in *Ube3a*^*m–/p+*^ mice at low stimulus durations. **(E)** Eating time is not correlated with OAIO. **(F)** Response latency is not correlated with OAIO during SD 16 sessions. **(G)** Response latency is not correlated with OAIO during SD1 sessions.

When the stimulus duration was 1 s, omissions after correct trials (OAC) occurred on >90% of trials in both WT and *Ube3a*^*m–/p+*^ groups (Figure 5A). OAC were greater in *Ube3a*^*m–/p+*^ mice than in WT littermates [Figure 5A; main effect of genotype: *F*_(1,__28)_ = 17.24, *p* = 0.0003]. *Ube3a*^*m–/p+*^ mice took significantly longer than WT littermates to eat pellet rewards [Figure 5B; *t*_(26)_ = 4.234, *p* = 0.0003]. Eating time was strongly correlated with OAC (Figure 5C; *R*^2^ = 0.7716, *p* < 0.0001). Therefore, we conclude that increased omissions in *Ube3a*^*m–/p+*^ mice are confounded by delays in eating rewards from prior trials.

To remove the confound of delayed pellet eating in *Ube3a*^*m–/p+*^ mice, we analyzed omissions on trials that did not follow a correct trial (omissions after incorrect/omission; OAIO). On these trials, no reward is present from the prior trial. OAIO were increased in *Ube3a*^*m–/p+*^ mice relative to WT littermates [Figure 5D; main effect of genotype: *F*_(1,__28)_ = 9.156, *p* = 0.0053]. Interestingly, increases in OAIO in *Ube3a*^*m–/p+*^ mice emerged as the stimulus duration decreased [Figure 5D; genotype × SD interaction: *F*_(5,__140)_ = 6.008, *p* < 0.0001]. OAIO were not correlated with eating time (Figure 5E; *R*^2^ = 0.03134, *p* = 0.3675) or response latency (Figures 5F,G; *R*^2^ = 0.02314, *p* = 0.4222 and *R*^2^ = 0.01947, *p* = 0.4621), but were correlated with performance during operant acquisition (Supplementary Figure 5). Expanding the definition of OAIO to also exclude trials following a (correct + omission) sequence did not meaningfully affect results (Supplementary Figure 6). OAIO and pellet eating phenotypes remained statistically robust in *Ube3a*^*m–/p+*^ mice after controlling for the age of animals (Supplementary Figure 7), but statistically meaningful group differences in OAIO were not observed during vSD and vITI test days (Supplementary Figure 8). We conclude that OAIO represent an alternative measure of omissions that is impaired in *Ube3a*^*m–/p+*^ mice and is not confounded by motor impairments or eating delays.

## Discussion

We used the 5CSRTT to test the hypothesis that attentional behavior is impaired in *Ube3a*^*m–/p+*^ mice. *Ube3a*^*m–/p+*^ mice displayed increased omissions during 5CSRTT training (Figure 4D), suggesting impaired attentional performance. However, *Ube3a*^*m–/p+*^ mice were also hypoactive during habituation to the testing environment (Figure 2C). Hypoactivity has been widely reported in *Ube3a*^*m–/p+*^ mice (Allensworth et al., 2011; Huang et al., 2013; Born et al., 2017; Sonzogni et al., 2018), and must be considered as a potential confound in any behavioral test using this line. Despite hypoactivity during habituation, *Ube3a*^*m–/p+*^ mice had normal response latency during the 5CSRTT (Figures 4F,G). In addition, response latency did not correlate with omissions within individual sessions (Figures 4H,I). Thus, we conclude that hypoactivity is not the cause of increased omissions in *Ube3a*^*m–/p+*^ mice. Rather, increased omissions in *Ube3a*^*m–/p+*^ were driven partially, but not fully, by delays in eating rewards from prior trials (Figure 5B). *Ube3a*^*m–/p+*^ mice averaged 15.6 ± 1.0 s to retrieve and eat rewards, whereas wild-type littermates averaged 10.4 ± 0.6 s. We used an intertrial interval of 5 s (Loos et al., 2009; Koike et al., 2016); thus, omissions on trials immediately following correct trials (Figure 5A) likely reflected when animals were eating rewards. By isolating trials where no prior reward was present (OAIO; omissions after incorrect or omission), we dissociated omissions from confounding eating delays in the *Ube3a*^*m–/p+*^ mouse model. Using this approach, we reported a significant interaction between stimulus duration and omissions during 5CSRTT training: omissions were increased only with shorter stimulus durations (Figure 5D). This result suggests that increased omissions may emerge in *Ube3a*^*m–/p+*^ mice as the attentional demand of the task increases.

Behavioral performance on the 5CSRTT demonstrates the need to carefully control for motor impairments when studying *Ube3a*^*m–/p+*^ mice. While gross motor hypoactivity did not drive omissions, we were surprised to find that *Ube3a*^*m–/p+*^ mice took longer to eat pellet rewards than wild-type littermates (Figure 5). It is unlikely that this delay is caused by a lack of motivation or the salience of reward, as *Ube3a*^*m–/p+*^ and WT mice earned comparable rewards during unrestricted magazine training (Figure 2) and completed a comparable number of trials during both operant acquisition and visuospatial discrimination (Figure 3). However, we did not explicitly test motivation (e.g., using a progressive ratio test) in the animals used for this study. It is also unlikely that this delay is caused by eating more of the reward pellet: all mice typically ate the entire pellet. Pellet eating time was defined by video analysis as time elapsed from initial head poke into the food magazine to retrieve reward until the reward was fully eaten or dropped. Thus, we hypothesize that the likeliest explanation for increased pellet eating time in *Ube3a*^*m–/p+*^ mice is impaired fine motor function (relevant for extracting and holding the pellet) and/or impairments in swallowing and chewing. Swallowing and chewing issues have been reported in individuals with AS (Varela et al., 2004; Glassman et al., 2017), but it is not known whether the *Ube3a*^*m–/p+*^ mouse model recapitulates these features. Unfortunately, the video resolution during this study was not sufficient to precisely dissect the cause of the increased pellet eating time observed in *Ube3a*^*m–/p+*^ mice. Further investigation is needed to evaluate if swallowing dysfunction, mouth malformations, and/or fine motor impairments are present in the *Ube3a*^*m–/p+*^ mouse model.

Future work using the 5CSRTT may consider adjusting task parameters to account for delayed eating time in *Ube3a*^*m–/p+*^ mice, regardless of its underlying cause. First, extension of the intertrial interval beyond 5 s could enable all mice to finish eating rewards before the start of the next trial. A 20–30 s intertrial interval would allow mice to finish eating rewards on most trials (Figure 5B). Alternatively, water rewards could be used instead of pellet rewards (Birtalan et al., 2020). Additionally, the 5CSRTT can be automated to allow *ad libitium* 24 h access to the task *via* a tube connecting the homecage to the testing chamber (Remmelink et al., 2017; Bruinsma et al., 2019). Self-paced 5CSRTT protocols require an active nosepoke into the empty food magazine to initiate trials, eliminating potential confounds related to pellet eating time on prior trials.

Attentional impairments are common in children with AS, typically manifesting as a short attention span (Tan et al., 2011; Sadhwani et al., 2019). The 5CSRTT provides a tool to assess attentional improvement following drug treatment in rodent models of AS. Typically, attention is assessed on a 5CSRTT test day with variable stimulus duration (Asinof and Paine, 2014). Here, we report a trend toward increased omissions (Supplementary Figure 3, *p* = 0.06) and OAIO (Supplementary Figure 7, *p* = 0.07) in *Ube3a*^*m–/p+*^ mice during the vSD test day, but these potential differences were not statistically meaningful. Instead, we report group differences in omissions during the 5CSRTT training phase. Impaired attentional performance is thus one of several potential explanations for the increased OAIO seen in *Ube3a*^*m–/p+*^ mice. For example, it is possible that *Ube3a*^*m–/p+*^ mice have difficulty achieving high rates of operant responding or that they have impairments in behavioral flexibility that are separate from attention. For future 5CSRTT studies in *Ube3a*^*m–/p+*^ mice with optimized task parameters, the vSD test day is likely the most appropriate place to assess true attentional performance. Beyond the 5CSRTT, other tasks, such as the continuous performance task, could also be used to assess attentional processing while engaging prefrontal circuits in *Ube3a*^*m–/p+*^ mice (Kim et al., 2015; Cope and Young, 2017; Hvoslef-Eide et al., 2018).

We hypothesize that the 5CSRTT may be used in the future as a behavioral readout of prefrontal circuit function in *Ube3a*^*m–/p+*^ mice. In rodents, the activity of ACC, a prefrontal subregion, is tightly linked to omissions on the 5CSRTT (Chudasama et al., 2003; Koike et al., 2016; Norman et al., 2021), though other circuits beyond ACC also contribute to attentional processing on the 5CSRTT and related tasks (Chudasama et al., 2012; Aoki et al., 2015; Kim et al., 2016; Wulaer et al., 2020). Future work may test the hypothesis that manipulating *Ube3a* levels in ACC neurons will selectively affect omissions on the 5CSRTT. More broadly, existing behavioral assessments in *Ube3a*^*m–/p+*^ mice are robust and reliable (Sonzogni et al., 2018), but lack test(s) that are driven primarily by prefrontal circuits. Developing readouts of prefrontal function in mouse models of AS will be critical to evaluate the overall effectiveness of treatments. We propose that the 5CSRTT represents an effective way to assess attention while engaging prefrontal circuits.

In addition to increased omissions, *Ube3a*^*m–/p+*^ mice also displayed behavioral phenotypes on other phases of the 5CSRTT task. Some of these differences, such as delayed operant acquisition (Figure 3C) and increased perseveration (Figures 3I,J), have been previously reported in *Ube3a*^*m–/p+*^ mice (Sidorov et al., 2018). Surprisingly, *Ube3a*^*m–/p+*^ mice reached visuospatial discrimination learning criteria faster than WT littermates (Figure 3F). This result implies that *Ube3a*^*m–/p+*^ mice were faster learners. The primary criterion used to assess visuospatial discrimination was accuracy, defined as ([correct responses]/[correct responses + incorrect responses]). *Ube3a*^*m–/p+*^ mice had a similar amount of correct responses, but had fewer incorrect responses, driving this delay in reaching criteria (Supplementary Figure 1). We hypothesize that this decrease in incorrect responses may be related to our prior finding that *Ube3a*^*m–/p+*^ mice have exaggerated operant extinction (Sidorov et al., 2018). While visuospatial discrimination (one light on, changing each trial) is typically considered a test of cognitive flexibility, it may also be interpreted as extinction of a prior rule (all lights on, poke any to receive reward). In this context, fewer incorrect responses would align with our prior finding of exaggerated operant extinction in *Ube3a*^*m–/p+*^ mice.

A limit to this study was the sole use of male mice for 5CSRTT experiments. While sex differences in 5CSRTT omissions would not be expected in wild-type mice (Papaleo et al., 2012; Ciampoli et al., 2017; Grissom and Reyes, 2019), sex differences have been reported for certain behaviors in *Ube3a*^*m–/p+*^ mice (Sonzogni et al., 2018; Koyavski et al., 2019). In addition, *Ube3a*^*m–/p+*^ mice may display sex differences in their responsiveness to environmental enrichment as a treatment in certain behavioral domains (Cosgrove et al., 2022). Future work using a modified 5CSRTT is needed to explicitly assess the role of sex on task performance in *Ube3a*^*m–/p+*^ mice. Another potential challenge in the interpretation of results is that *Ube3a*^*m–/p+*^ mice were, on average, younger than WT controls (Supplementary Figure 8A). This difference occurred by chance due to the Mendelian inheritance of the mutant *Ube3a* allele (Supplementary Figure 9). By using age as a covariate for statistical analysis, we confirmed that age differences between groups did not affect our main behavioral findings (Supplementary Figure 8C). While not planned in this case, the group difference in age was beneficial in that it enabled us to study behavior in *Ube3a*^*m–/p+*^ mice in groups that were weight-matched (Supplementary Figure 8B). Increased weight has been widely reported in *Ube3a*^*m–/p+*^ mice (van Woerden et al., 2007; Huang et al., 2013; Born et al., 2017; Judson et al., 2017, 2021; Sonzogni et al., 2018; Wolter et al., 2020), and weight differences have the potential to confound behavioral tests. Here, behavioral phenotypes were present on the 5CSRTT in *Ube3a*^*m–/p+*^ mice that were the same weight as WT controls. Future behavioral studies using *Ube3a*^*m–/p+*^ mice may consider weight matching adult mice instead of age matching.

Overall, the 5CSRTT can be used to assess attention and impulsivity in *Ube3a*^*m–/p+*^ mice and can be optimized in the future to account for other behavioral impairments in this mouse model. The 5CSRTT can be used in conjunction with existing behavioral assessments to extend the range of testing to include more complex tasks that are likely to be regulated by prefrontal circuits.

## Materials and methods

### Animals

All methods were carried out in accordance with relevant guidelines and regulations. Procedures were approved by the Institutional Animal Care and Use Committee of the University of North Carolina at Chapel Hill. Mice were group housed on a 12 h light/dark cycle. Experimental AS model mice (*Ube3a*^*m–/p+*^) (Jiang et al., 1998) and wild-type littermates (WT; *Ube3a*^*m+/p+*^) on a C57BL/6J congenic background were generated by crossing wild-type males and females with paternal *Ube3a* inheritance (*Ube3a*^*m+/p–*^). Mice were genotyped using polymerase chain reaction (PCR) using the following primers: WT Forward (5′-GCTCAAGGTTGTATGCCTTGGTGCT-3′), Mutant Forward (5′- TGCATCGCATTGTGTGAGTAGGTGTC), and WT reverse (5′-ACTTCTCAAGGTAAGCTGAGCTTGC- 3′). Adult male mice (∼P70-P160 at the beginning of study) were used for behavioral experiments (WT mean: 136 ± 6 days, *Ube3a*^*m–/p+*^ mean: 109 ± 8 days). A table with information on all breeders and experimental mice is included in Supplementary Figure 9.

### Behavioral equipment

We used modular operant conditioning chambers (MED-Associates, ENV-307 W) equipped with five response apertures on one wall and a food magazine on the opposite wall. A chamber light over the magazine illuminated the whole chamber. All chambers were placed in sound-attenuating ventilated cubicles. The response apertures and magazine contained yellow LED stimulus lights and infrared response detectors. Stimulus lights inside the response apertures were controlled individually to provide visual cues as noted.

### Five-choice serial reaction time task

We performed the 5CSRTT based on well-established protocols (Pattij et al., 2007; Loos et al., 2009; Koike et al., 2016) with minor modifications. Sample sizes (*n* = 14–16 mice per group) were determined *a priori* by availability of mice. Food restricted mice performed multiple stages of the task sequentially: habituation, magazine training, operant acquisition, visuospatial discrimination, 5CSRTT training, and 5CSRTT testing (Figure 1). At each stage, mice performed a single session per day. Testing occurred 7 days a week during the light phase at the same time each day. For the duration of experiments, mice received 2 h of unrestricted feeding immediately after testing with *ad libitum* access to water. Mice had *ad libitum* access to water in their home cage at all times. During the task, 20 mg dustless precision pellets (BioServ) were delivered as rewards where noted.

### Habituation

Mice were habituated for 25 min inside the behavioral chamber for one session. During habituation, only the house light was on, and no rewards were delivered. Motion was recorded using a camera above the arena (Logitech) and tracked manually using Tracker video analysis software.

### Magazine training

Magazine training consisted of sessions on two consecutive days where pellets were delivered into the food magazine with pseudorandom intertrial intervals (ITIs) of 4, 8, 16, and 32 s. Pellet delivery coincided with switching on the magazine light. Retrieval of a pellet initiated the next trial. The magazine light was off during the ITI. Sessions lasted until mice had retrieved 50 pellets or 25 min, whichever came first.

### Operant acquisition

Operant acquisition trials began by illuminating the chamber light and all five stimulus apertures. The chamber light remained on for the entire session. A response into any of the five illuminated apertures turned off all the stimulus lights, switched on the magazine light, and delivered a food pellet reward. A trial, defined as a correct response and retrieval of pellet from the magazine, was considered complete when the pellet was retrieved, at which time the magazine light would turn off and the aperture lights would turn back on. Sessions lasted 60 trials or 25 min, whichever came first (Loos et al., 2009). Operant acquisition was considered complete, and mice advanced to visuospatial discrimination, after performing > 25 trials in two consecutive sessions. Mice were “primed” in order to train them to nose poke sufficiently deep into the stimulus apertures to trigger the infrared beam and register a poke. Priming consisted of pellets placed in each of five apertures on the first 2 days of operant acquisition. If less than 5 trials occurred in a subsequent session, mice were primed the following day.

### Visuospatial discrimination

Trials began by illuminating the chamber light and only one of the five stimulus apertures. The illuminated aperture varied randomly on a trial-by-trial basis. A response into the illuminated aperture (“correct response”) switched off the stimulus light, switched on the magazine light, and triggered the delivery of a reward. Reward retrieval initiated an ITI of 5 s before the onset of the next trial. Sessions lasted 60 trials or 25 min, whichever came first (Loos et al., 2009). An incorrect response was defined as a response into a non-illuminated aperture. To advance to 5CSRTT training, mice had to reach criteria of > 25 trials and > 50% accuracy (defined as correct responses/[correct responses + incorrect responses]) in two consecutive sessions.

### Five-choice serial reaction time task training

Trials (trial structure illustrated in Figure 4B) were similar to visuospatial discrimination, except visual stimuli were presented with a fixed stimulus duration (SD). Correct responses occurred and rewards were delivered if the mouse responded in the illuminated aperture either when the aperture was illuminated or in the 4 s limited hold period after the light turned off. Incorrect responses into a non-illuminated aperture, premature responses, and omissions resulted in a 5 s time-out period, during which all stimulus lights and chamber lights were turned off (Pattij et al., 2007; Loos et al., 2009). The SD remained constant throughout each individual session, and the ITI (5 s) was constant across all sessions. Mice began with a 16 s SD which was gradually decreased in subsequent sessions to 8, 4, 2, 1.5, and 1 s as the subject reached pre-determined criteria (< 30% omissions, > 60% accuracy, > 50 trials) in a single session or after 10 sessions at the same SD if mice did not reach criteria (Figure 1). Sessions lasted 30 min or 100 trials, whichever came first.

### Five-choice serial reaction time task testing

5CSRTT testing sessions had a similar trial structure to 5CSRTT training, except that either the SD or ITI varied pseudorandomly within a single session. We performed 2 days of testing, with a variable SD to test attention (Day 1) and a variable ITI to test impulsivity (Day 2) (Figure 1). On Day 1, sessions for test days lasted 30 min or 100 trials, whichever came first. The attentional load was increased by manipulating the SD (1 s; 0.5 s; 0.2 s). On Day 1, the ITI was 5 s, the limited hold was 4 s, and the time out was 5 s. On Day 2, sessions lasted 45 min or 100 trials, whichever came first. Inhibitory control was increased by shortening the manipulating the ITI (5 s; 7.5 s; 12.5 s). On Day 2, the SD was 1 s, the limited hold was 4 s, and the time out was 5 s.

### Data analysis

To assess perseveration, we first rank-ordered the five illuminated apertures based on the number of responses into each aperture during operant acquisition (Figure 3I). Next, we carried over these rankings and assessed the number of errors that were made in each aperture during the visuospatial discrimination phase (Figure 3J). This approach (Krueger et al., 2011; Sidorov et al., 2018) allowed us to ask both whether *Ube3a*^*m–/p+*^ mice have increased perseveration, and whether this initial perseveration results in errors later on subsequent phases of the task. During 5CSRTT training and testing, response latency was defined as the amount of time from visual cue onset to nosepoke in either the correct or incorrect aperture. Omissions after correct responses (OAC; Figure 5A) were defined as the percentage of omissions on trials immediately following a correct trial. Omissions after incorrect responses or omissions (OAIO; Figure 5D) were defined as the percentage of omissions of trials immediately following an incorrect trial or an omission. Eating time was defined as the amount of time that elapsed from initial head poke to retrieve pellet until the pellet was eaten or dropped. Eating time analysis in Figure 5B was performed in the final session of 5CSRTT training with a SD of 16 s. Two videos at this stage from WT mice were corrupted; thus, *n* = 12 for WT in Figure 5B.

### Statistics

Reported “n” represents animals, and no animals were excluded from behavioral analysis. All error bars indicate ± SEM. Experimenters were blind to genotype and all studies were performed using littermate controls. Student’s *t*-tests were used in Figures 2C–E, 3C,D,F–H, 5B and Supplementary Figures 8A,B. Two-way ANOVA was used for Figures 3I,J, and Two-way RM ANOVA was used in Figures 4C,D,F,G, 5A,D and Supplementary Figures 1B,C, 2A–C, 3B–D,F–H, 4B, 6A,B, 8A,B, and two-way ANOVA was used in Figures 3I,J. For ANOVAs, *post hoc* Bonferroni tests were used when there was a main effect of genotype or interaction between genotype and the second factor. Linear regression was used in Figures 4E,H,I, 5C,E–G and Supplementary Figures 5, 7C. Age as a covariate was added in all models in Supplementary Figure 7D using the appropriate lm() or lmer() function in R. Statistical tests were performed using GraphPad Prism 9 and R.

## Data availability statement

The original contributions presented in this study are included in the article/Supplementary material, further inquiries can be directed to the corresponding author/s.

## Ethics statement

The animal study was reviewed and approved by the Institutional Animal Care and Use Committee of the University of North Carolina at Chapel Hill.

## Author contributions

MS and PN-M contributed to conception and design of the study and wrote the first draft of the manuscript. PN-M performed behavioral studies. PN-M, DD, CG, and MS contributed to data analysis and statistical analysis and contributed to sections of the manuscript. All authors contributed to manuscript revision, read, and approved the submitted version.
