# Supplementary material for: Dissociating motor impairment from five-choice serial reaction time task performance in a mouse model of Angelman syndrome
Source: Front Behav Neurosci. 2022 Sep 23;16:968159. doi: 10.3389/fnbeh.2022.968159 (PMC9539753; doi:10.3389/fnbeh.2022.968159)
Supplement: Supplementary file 1 [file Data_Sheet_1.PDF]

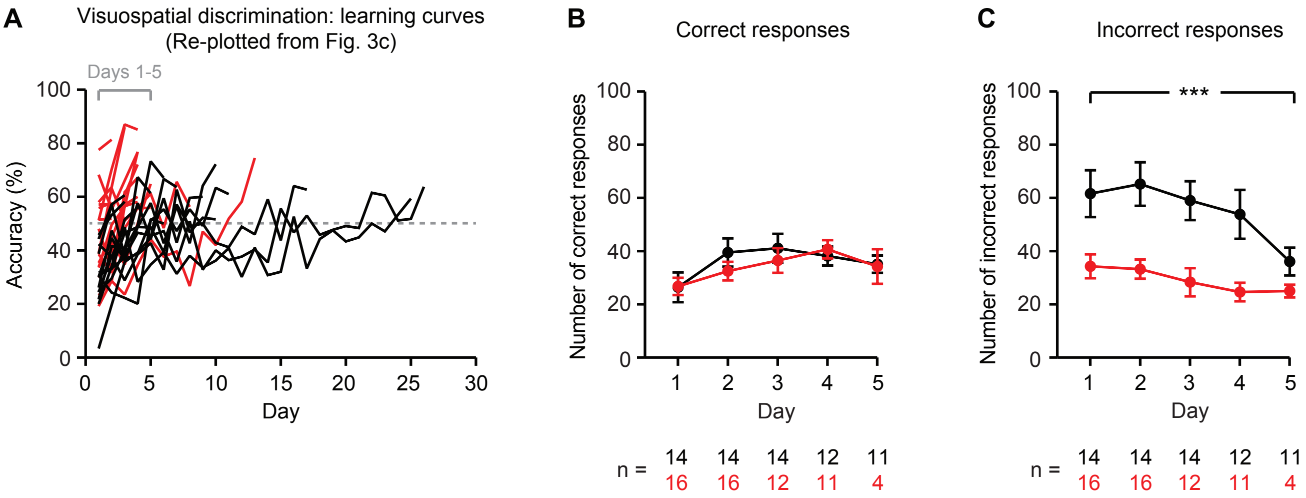

Supplementary Figure 1: *Ube3a$^{m-/p+}$* mice reach visuospatial discrimination criteria faster because they have fewer incorrect responses. WT: black, *Ube3a$^{m-/p+}$* (AS): red. (a) Figure 3c is re-plotted here for reference. Days 1-5 of visuospatial discrimination training are shown in panels B-C. Beyond Day 5, sample size drops off substantially due to mice reaching criteria. (b) WT and *Ube3a$^{m-/p+}$* mice have a similar amount of correct responses over the first five days of visuospatial discrimination training (mixed-effects analysis; main effect of genotype: $F_{(1,28)} = 0.006158$, $p = 0.9380$; genotype X day interaction: $F_{(4,86)} = 1.454$, $p = 0.2233$). (c) *Ube3a$^{m-/p+}$* mice have fewer incorrect responses than WT controls over the first five days of visuospatial discrimination training (main effect of genotype: $F_{(1,28)} = 16.02$, $***p = 0.0004$; genotype X day interaction: $F_{(4,86)} = 0.6205$, $p = 0.6491$).

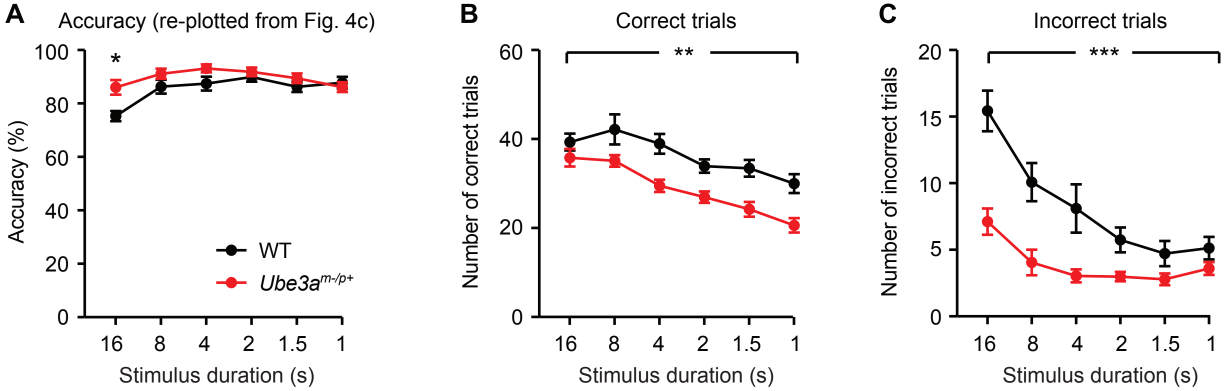

Supplementary Figure 2: Increased accuracy in *Ube3a*$^{m-/p+}$ mice during early stages of 5CSRTT training results from fewer incorrect trials. WT: black, *n* = 14. *Ube3a*$^{m-/p+}$ (AS): red, *n* = 16. (a) Figure 4c is re-plotted here for reference. (b) *Ube3a*$^{m-/p+}$ mice have fewer correct trials than WT controls (two-way RM ANOVA; main effect of genotype: $F_{(1,28)}$ = 11.29, ***p* = 0.0023; genotype X SD interaction: $F_{(5,140)}$ = 2.135, *p* = 0.0648). (c) *Ube3a*$^{m-/p+}$ mice have fewer incorrect trials than WT controls (two-way RM ANOVA; main effect of genotype: $F_{(1,28)}$ = 17.25, ****p* = 0.0003). The disparity in incorrect trials is more pronounced with a longer stimulus duration (genotype X SD interaction: $F_{(5,140)}$ = 6.349, *****p* < 0.0001).

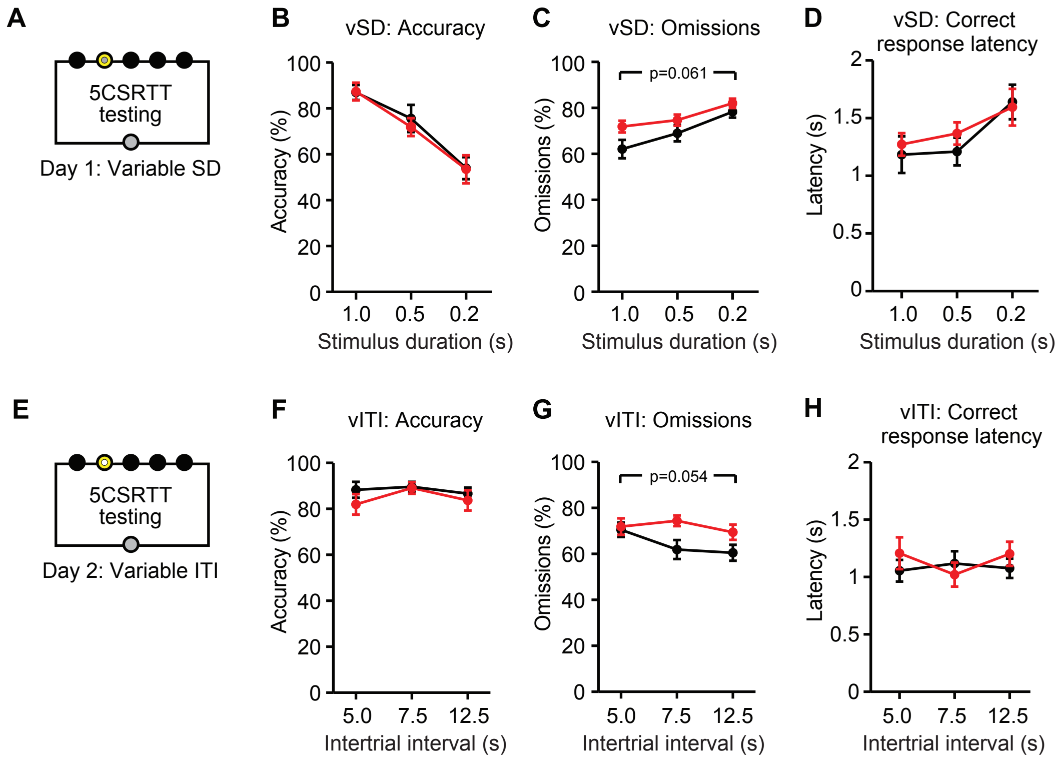

Supplementary Figure 3: *Ube3a$^{m-/p+}$* mice trend towards increased omissions during 5CSRTT testing. WT: black, $n = 14$. *Ube3a$^{m-/p+}$* (AS): red, $n = 16$. (a-d) Performance of mice on Day 1 of 5CSRTT testing, with variable stimulus duration (vSD). (a) Schematic. (b) Accuracy does not differ by genotype ($F_{(1,28)} = 0.1031$, $p = 0.7505$). (c) There is a trend towards increased omissions in *Ube3a$^{m-/p+}$* mice across short stimulus durations ($F_{(1,28)} = 3.824$, $p = 0.0606$). (d) Correct response latency does not differ by genotype ($F_{(1,28)} = 0.2703$, $p = 0.6072$). (e-h) Performance of mice on Day 2 of 5CSRTT testing, with variable intertrial interval (vITI). (e) Schematic. (f) Accuracy does not differ by genotype ($F_{(1,28)} = 0.9955$, $p = 0.3269$). (g) There is a trend towards increased omissions in *Ube3a$^{m-/p+}$* mice across intertrial intervals ($F_{(1,28)} = 4.055$, $p = 0.0538$). (h) Correct response latency does not differ by genotype ($F_{(1,28)} = 0.4175$, $p = 0.5234$).

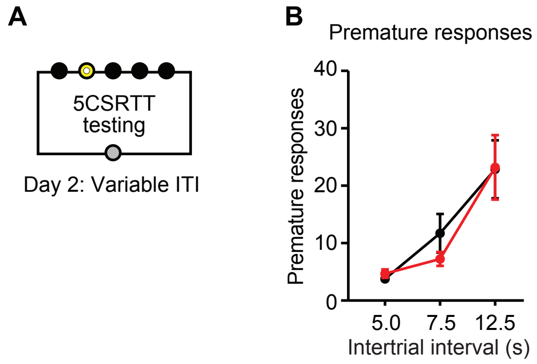

Supplementary Figure 4: *Ube3a$^{m-/p+}$* mice have normal impulsivity. WT: black, *n* = 14. *Ube3a$^{m-/p+}$* (AS): red, *n* = 16. (a) Schematic Day 2 of 5CSRTT testing with variable intertrial interval (ITI) to test impulsivity. (b) Premature responses do not differ by genotype ($F_{(1,28)} = 0.09773$, $p = 0.7569$).

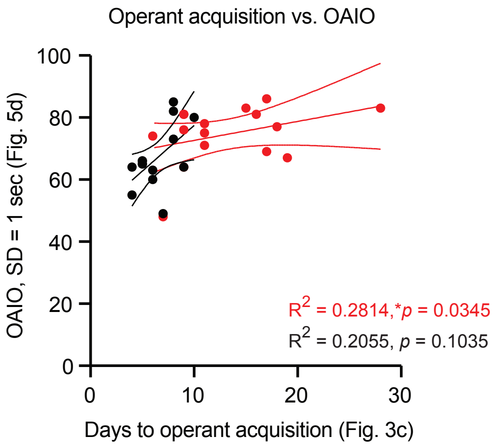

Supplementary Figure 5: Omissions after incorrect trials or omissions (OAIO) are correlated with operant acquisition in *Ube3a*$^{m-/p+}$ mice. WT: black, *n* = 14. *Ube3a*$^{m-/p+}$ (AS): red, *n* = 16.

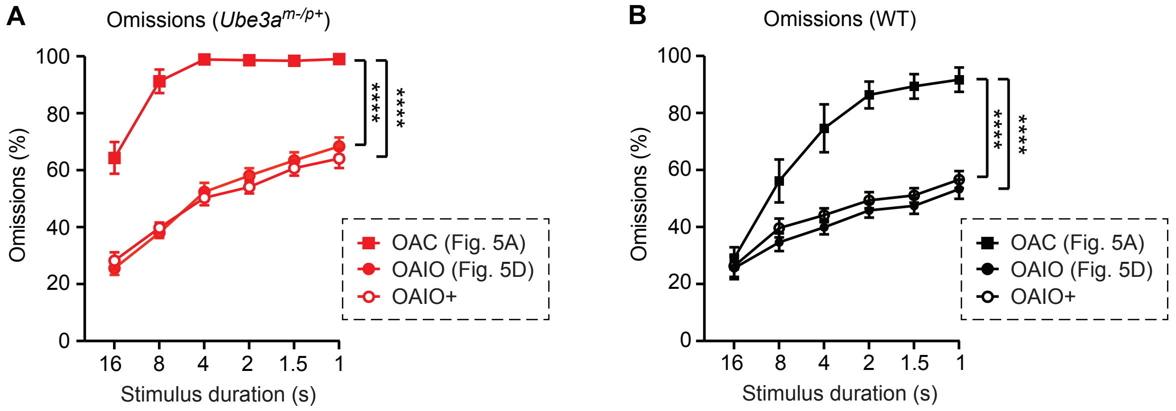

Supplementary Figure 6: OAIO are similar if trials following a (correct + omission) sequence are excluded from analysis. OAIO+ (open circles) is defined as omissions on trials that (1) do not follow a correct trial, and (2) do not follow a (correct + omission) sequence. (a) *Ube3a*$^{m-/p+}$ mice (n = 16) had more OAC (squares) than either OAIO (closed circles) or OAIO+ (open circles) (two-way RM ANOVA; main effect of analysis method: $F_{(2,30)} = 787.1$, $p < 0.0001$; post hoc OAC vs. OAIO: ****$p < 0.0001$; post hoc OAC vs. OAIO+: ****$p < 0.0001$), but there was no statistical difference between OAIO and OAIO+ (post hoc OAIO vs. OAIO+: $p = 0.4581$). (b) WT mice (n = 14) had more OAC (squares) than either OAIO (closed circles) or OAIO+ (open circles) (two-way RM ANOVA; main effect of analysis method: $F_{(2,26)} = 41.22$, $p < 0.0001$; post hoc OAC vs. OAIO: ****$p < 0.0001$; post hoc OAC vs. OAIO+: ****$p < 0.0001$), but there was no statistical difference between OAIO and OAIO+ (post hoc OAIO vs. OAIO+: $p = 0.6191$).

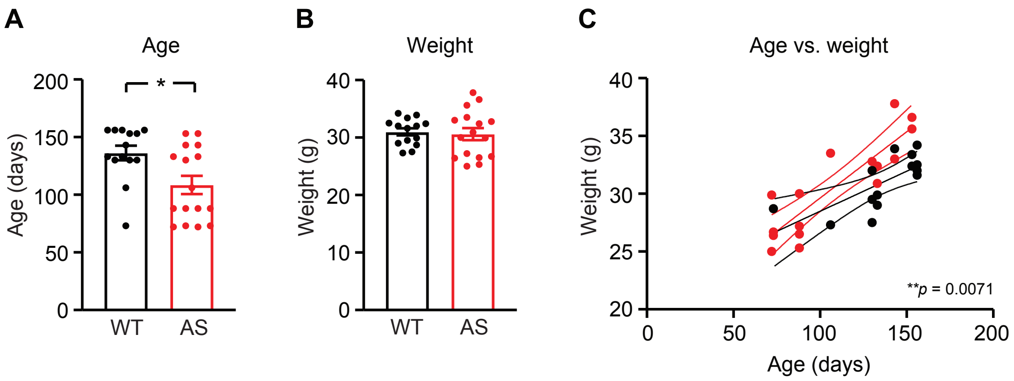

D

Behavioral test	Figure	Test used	Result (with age as covariate)
Distance traveled (habituation)	2c	Student's t-test	****$p < 0.0001$
Days to criteria (operant acquisition)	3c	Student's t-test	***$p = 0.000551$
Days to criteria (visuospatial discrimination)	3f	Student's t-test	$p = 0.0836$
Perseveration (acquisition)	3i	Two-way ANOVA	**$p = 0.00879$ (interaction)
Perseveration (discrimination)	3j	Two-way ANOVA	****$p < 0.0001$ (interaction)
Pellet eating time (5CSRTT training)	5b	Student's t-test	***$p = 0.000432$
OAIO (5CSRTT training)	5d	Two-way RM ANOVA	***$p = 0.000632$ (genotype)<br>**$p = 0.00334$ (interaction)

Supplementary Figure 7: 5CSRTT phenotypes in *Ube3a*$^{m-/p+}$ mice persist when accounting for age. WT: black, *Ube3a*$^{m-/p+}$ (AS): red. (a) *Ube3a*$^{m-/p+}$ mice used for experiments were younger than WT littermates (Student's t-test: *$p = 0.0116$). (b) Weight was not different by genotype ($p = 0.7555$). (c) When controlling for age, *Ube3a*$^{m-/p+}$ mice did show increased weight as expected (**$p = 0.0071$). (d) Re-assessing main behavioral phenotypes in *Ube3a*$^{m-/p+}$ mice while accounting for age of animals. All phenotypes tested remained statistically significant except visuospatial discrimination when accounting for age.

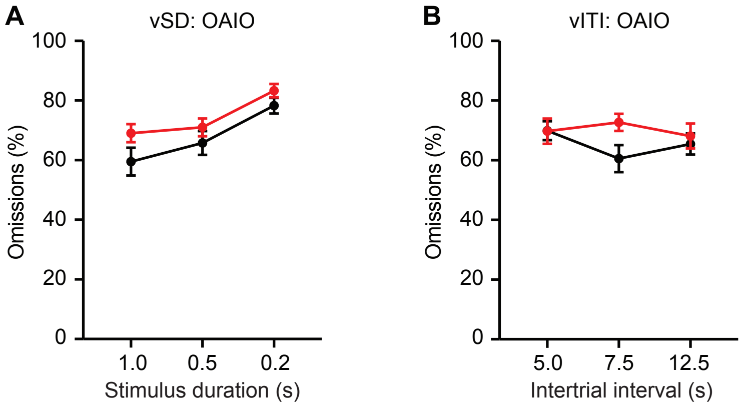

Supplementary Figure 8: OAIO during 5CSRTT testing sessions. WT: black, *n* = 14. *Ube3a*$^{m-/p+}$ (AS): red, *n* = 16. (a) OAIO during the 5CSRTT test day with variable stimulus duration (vSD). Two-way RM ANOVA, main effect of genotype: $F_{(1,28)}$ = 3.463, *p* = 0.0733. (b) OAIO during the 5CSRTT test day with variable intertrial interval (vITI). Two-way RM ANOVA, main effect of genotype: $F_{(1,28)}$ = 1.462, *p* = 0.2368.

Breeder ID	Litter ID	Mouse ID	Age (days)	Genotype
1	1-1	1583	156	WT
1	1-1	1584	156	WT
1	1-1	1585	156	WT
1	1-1	1586	156	WT
1	1-2	1598	133	WT
1	1-2	1599	133	AS
1	1-2	1600	133	AS
1	1-2	1601	133	WT
1	1-3	1579	88	AS
1	1-3	1580	88	AS
1	1-3	1581	88	AS
1	1-3	1582	88	AS
2	2-1	1571	153	AS
2	2-1	1572	153	WT
2	2-1	1573	153	AS
2	2-1	1574	153	WT
2	2-2	1591	130	WT
2	2-2	1592	130	WT
2	2-2	1593	130	WT
2	2-2	1594	130	AS
3	3-1	1595	143	AS
3	3-1	1596	143	WT
3	3-1	1597	143	AS
3	3-2	1575	72	AS
3	3-2	1576	72	AS
4	4-1	1569	106	AS
4	4-1	1570	106	WT
5	5-1	1587	73	AS
5	5-1	1588	73	AS
5	5-1	1590	73	WT

Supplementary Figure 9: Mice used for experiments. A total of five breeding pairs (wild-type male X *Ube3a*$^{m+/p-}$ female) generated nine litters used for this study.
